# Tissue resident memory T cells create genetically distinct immune microenvironments within melanoma metastasis

**DOI:** 10.1186/2051-1426-3-S2-O15

**Published:** 2015-11-04

**Authors:** Kavita Dhodapkar, Noffar Bar, Chandra Sekhar Boddupalli, Krishna Kadaveru, Zifeng Mai, Yanhong Deng, Mario Sznol, Madhav Dhodapkar

**Affiliations:** 1 Yale University, New Haven, CT, USA; 2 Yale University School of Medicine, New Haven, CT, USA

## 

Blockade of inhibitory immune checkpoints (ICPs) improves survival in melanoma patients. Interestingly, the expression of these ICPs in most tumor tissues including melanoma is restricted to only a subset of tumor-infiltrating immune cells (TIICs). Here, we utilize single cell mass cytometry (CyTOF), functional profiling and TCR sequencing of freshly isolated cells to evaluate the phenotypic, functional and genetic heterogeneity of TIICs in patients (n=43) with advanced melanoma to define the subsets of immune cells involved in ICP-mediated regulation.

Using paired blood and tumor samples from melanoma patients and CyTOF analysis with 36 different markers, we show that T and myeloid cells within tumor metastases are distinct from their circulating counterparts. T cells within metastases are highly enriched for CD45RO+, CD69+, CD103+ tissue resident CD8+ memory T cells (T_RM_). Expression of some ICPs (such as PD-1, Tim3 and PD-L1) in TIICs is primarily restricted to T_RM_ cells and to CD16+ subset of inflammatory/patrolling myeloid cells. Functional analysis of freshly isolated tumor T cells using multiplex luminex ELISA revealed that despite expression of multiple ICPs, tumor-associated T cells are functional, but have an altered cytokine profile compared to circulating T cells. In contrast to blood T cells, tumor infiltrating T cells secrete lower levels of IL-2, IFN-γ and TNF-α, but comparable levels of IL4, IL5 and IL17.

Tissue restriction of murine T_RM_ cells without recirculation has been extensively demonstrated in the form of lack of equilibrium in parabiotic mice that share a systemic circulation. The finding that melanoma metastatic lesions are enriched for T_RM_ cells suggested the possibility that each of the metastatic lesions may contain a distinct microcosm of T cells without equilibration, in spite of shared systemic circulation within the same host, analogous to the biology in parabiotic mice. In order to test this directly, we compared the TCR sequences of memory CD4 and CD8 T cells isolated from patients with biopsies of multiple metastatic sites. Using high throughput deep sequencing of rearranged TCRβ loci, we find that each biopsy site has a distinct TCR usage with only partial overlap with the other metastatic site from the same patient.

Together these data suggest that immunity to melanoma is highly regional and enriched for tumor-resident memory T cells creating genetically unique immune-microenvironments within each metastatic lesion. Regional nature of immunity within metastases has several implications for current strategies to harness and measure tumor immunity.

